# John Shaw Billings: A Memoir

**Published:** 1915-12

**Authors:** 


**John Shaw Billings: A Memoir.** By FIELDING H. GARRISON, M.D. Pp. ix. 432. New York and London: G. P. Putnam's Sons, The Knickerbocker Press. 1915. Price 10s. 6d.

"Dr. H. sees more sights than I, but I see more men than he." This remark, in one of Dr. Billings' letters to his wife when travelling through Europe studying the planning of the Johns Hopkins Hospital, seems to demonstrate in one sentence the kind of man he was. It offers the readiest explanation why Billings, despite his opportunities for growing "red-tape bound" in the Surgeon-General's Office, despite his life-long training in administration, and his later occupation as Chief Cataloguer to the medical world, nevertheless resisted the tendency to become one of Milton's "ferrets and mousehunts of an index."

Unlike the majority of men whose bent is towards record gathering and arrangement, John Shaw Billings was never obsessed by the beauty of mere orderliness. In his mind indexing, filing and cataloguing were only valuable in so far as they served the needs of men. His libraries, his hospitals and the University which he made and managed were not sights to be seen, they were instruments for men's use. In this respect Billings dominated his contemporaries. Probably no man who, to all intents and purposes, ceased at so early an age from the practice and craft of medicine, has ever exerted a like influence upon its destinies. Here was a man ready all his working days to carry out Trousseau's injunction to his students, "Rich in the possessions bequeathed to you by the physicians of the past, to you belongs the duty of uniting modern science with ancient wisdom, and of rekindling the temporarily despised torch of old medical traditions." Royally did Billings enter into this heritage, and realising its possibilities, administered and enjoyed it to the benefit of his fellow-man and his own great contentment.

Born in Indiana, schooled and taught as a farmer's son, at the age of ten or so his father moved his home to keep a village store as postmaster with a shoemaker's shop. In this environment Billings says of himself, "I was *omnivorous*, read everything in English as it came, philosophy, theology, natural science, history, travels and fiction ;" also "the Bible through verse by verse, *Robinson Crusoe, Deerslayer, Pathfinder* and *Pilgrim's Progress.*" "I had for my own *Robinson Crusoe, Marco Paul in the Forests of Maine, Harry and Lucy* and *Plutarch's Lives,* and was quite sure I did not want to be a farmer." With his father's encouragement he entered and graduated at Miami University, presently passing on to the Medical College of Ohio. The outbreak of the Civil War took Billings from his Demonstratorship of Anatomy at Ohio to be a military surgeon with the Army of the Potomac. A great part of this memoir is filled with letters from the war, written with a freedom of criticism which we, in this war of ours, read with some amazement, but without any regret that our own correspondents do not write with the same frankness.

It was during the course of the Civil War that Billings was relieved from duty in the field and assigned to the Washington branch of the Medical Director of the Army of the Potomac, to assist in collating the various field reports which went to furnish the Medical and Surgical History of the War. Afterwards he was transferred to the Surgeon-General's Office, and remained there until his retirement from the Army in 1895. Part of the work he did there will remain as a lasting monument in the shape of the *Index-Catalogue of the Surgeon-General's Library at Washington.*

Another outcome of his genius for organisation is the Johns Hopkins Hospital and Medical School. Of less interest perhaps to us, but a no less memorable achievement, was his work in connection with the New York Public Library.

But take him all round, we like best to think of Dr. J. S. Billings as the master-builder, who knew how to find men and secure them for his designs. He was at his greatest in judging material and using it, such material as Professor Welch, Osler, Halsted, Thayer and all that brilliant group who have made Baltimore world-famous ; our own Bristol student, Robert Fletcher, his coadjutor in the *Index-Catalogue* and the *Index Medicus,* and his able successor and biographer, Dr. Fielding H. Garrison.

As a judge of men Billings, aided not a little by medical training, and by physiological and psychological knowledge, towers above most of those statesmen or generals in whom the knowledge of men is commonly believed to reach its highest development. He was undoubtedly a great man, who delighted, unlike many lesser great ones, to surround himself with men of outstanding talents and ability, a true midwife of learning.

Dr. Garrison's biography of his chief does full justice to his memory, and will be read and re-read with increasing interest and satisfaction.

